# Transcriptome analysis of two contrasting rice cultivars during alkaline stress

**DOI:** 10.1038/s41598-018-27940-x

**Published:** 2018-06-25

**Authors:** Ning Li, Hualong Liu, Jian Sun, Hongliang Zheng, Jingguo Wang, Luomiao Yang, Hongwei Zhao, Detang Zou

**Affiliations:** Rice Research Institute, College of Agriculture, Northeast Agricultural University, Harbin, China

## Abstract

Soil alkalinity greatly affects plant growth and crop productivity. Although RNA-Seq analyses have been conducted to investigate genome-wide gene expression in response to alkaline stress in many plants, the expressions of alkali-responsive genes in rice have not been previously investigated. In this study, the transcriptomic data between an alkaline-tolerant (WD20342) and an alkaline-sensitive (Caidao) rice cultivar were compared under alkaline stress conditions. A total of 962 important alkali-responsive (IAR) genes from highly differentially expressed genes (DEGs) were identified, including 28 alkaline-resistant cultivar-related genes, 771 alkaline-sensitive cultivar-related genes and 163 cultivar-non-specific genes. Gene ontology (GO) analysis indicated the enrichment of IAR genes involved in various stimulus or stress responses. According to Kyoto Encyclopedia of Genes and Genomes (KEGG) pathway analysis, the IAR genes were related primarily to plant hormone signal transduction and biosynthesis of secondary metabolites. Additionally, among these 962 IAR genes, 74 were transcription factors and 15 occurred with differential alternative splicing between the different samples after alkaline treatment. Our results provide a valuable resource on alkali-responsive genes and should benefit the improvement of alkaline stress tolerance in rice.

## Introduction

Rice is one of the most important staple food crops and feeds more than two billion people worldwide^1–3^. Among the adverse environmental factors, soil salinization is a critical problem for rice growth and imposes major challenges to the productivity of rice^4^. Salinization affects negatively a variety of rice growth and development processes such as seedling growth, tillering, metabolism and transcription^5,6^. Currently, approximately 20% of the total paddy rice planting area is in the saline-alkali soil, and more seriously, the area of salinization is expanding in China^7^. In previous studies, saline-alkaline stress is divided into two categories: salt stress and alkaline stress^8^. Alkalization of soil due to NaHCO_3_ and Na_2_CO_3_ might more serious than soil salinization caused by neutral salts, such as NaCl and Na_2_SO_4_, in certain respects^9–11^. The alkalinity inhibits seed germination, plant growth and productivity by the osmotic stress and ion injury, and improves soil pH (>8.5)^12^. In the past, many researches have concentrated on how plants respond to alkaline stress. The high concentration of Na^+^, HCO^−^_3_, and CO^2−^_3_ in soils causes the increase in external osmotic pressure and ion imbalance in plants^13^. In contrast to salt stress, alkaline stress which is caused by high pH of alkaline soil inhibits plant growth by imposing adverse effect on roots, decreasing nutrient solubility, increasing organic acids imbalance, distribution and accumulation of inorganic ions, especially disrupting cellular pH stability^14^. Therefore, improving alkaline stress tolerance is essential for achieving high and stable yield in rice.

Rice is a model plant in the monocotyledons^15^. Many valuable achievements have increased the understanding about the molecular and cellular mechanisms of rice responding and tolerating adverse conditions in recent years^16–19^. With the rapid development of high-throughput technologies, microarray analyses have been conducted to identify stress-mediated differences in the level of gene expression. These studies revealed that many differentially regulated genes that are significantly associated with stress resistance under various conditions of abiotic stress^20–22^. In 2016, Shankar *et al*.^23^ compared the transcriptomes of Nagina 22 (drought-tolerant cultivar) and Pokkali (salinity-tolerant cultivar) with a susceptible cultivar (IR64) under control and stress conditions and found a total of 801 and 507 genes that were specifically differentially expressed in Nagina 22 and Pokkali under stress conditions, respectively. Additionally, Zhang *et al*.^24^ investigated the differentially expressed genes between upland and lowland rice cultivars under drought conditions using RNA-seq, and identified 436 genes that showed differential expression, which were classified into 8 categories. Moreover, Zhou *et al*.^25^ investigated the effect of salt stress on gene expression in Dongxiang wild rice leaves and root tissues by using Illumina HiSeq2000 platform and found that fewer salt-responsive genes responded in the roots than that in the leaves. In 2014, Shen *et al*.^26^ compared the genome-wide gene expression profiles of one cold-sensitive rice variety and three cold-tolerant rice varieties under both normal temperature and cold stress conditions and found a total of 2242 differentially expressed genes (DEGs), among all the cultivars. In 2015, Yang *et al*.^27^ performed comparative transcriptomes analysis of leaf sheaths and roots of rice in response to nitrogen-deficient and nitrogen-sufficient conditions by RNA-Seq, and identified a total of 1158 transcripts in the leaf sheaths and 492 ones in the roots that were differentially expressed in response to the nitrogen-deficiency. Although great advances have been achieved in the past few decades, to date, progresses are not sufficient toward the generation of stress-tolerant rice varieties and the understanding of the general molecular basis of stress-resistance, particularly for alkaline stress. Therefore, the exploration and the function prediction of alkali-response genes during alkaline stresses is the efficient approach for the molecular breeding of alkali-resistant rice cultivars.

Under alkaline stress, alkalinity-tolerant plants can sequestrate Na^+^ in vacuoles by compartmentalization of ions at the cellular level to enhance tolerance for high concentration of ions. For example, the alkaline-tolerant *indica* variety ‘WD20342’ held significantly the higher concentration of K^+^ and the lower concentration of Na^+^ in shoots and roots of seedlings under alkaline stress (0.15% Na_2_CO_3_, pH = 8.5) than the alkaline-susceptible *japonica* variety ‘Caidao’^28^ (for details, also see Supplementary Fig. S1).

In this study, we compared the transcriptomes of alkali-tolerant rice cultivar WD20342 and alkali-sensitive rice cultivar Caidao using RNA-seq analysis under control and alkaline stress conditions. The leaves of Caidao and WD20342 under control and stress conditions at the seedling stage were sequenced and differentially expressed genes (DEGs) and important alkali-responsive (IAR) genes were identified. Functional categorization of DEGs and IAR genes was conducted to reveal various metabolic pathways involved in responses to alkaline stress. Furthermore, the different alternative splicing of genes, novel transcripts and transcription factors in the two rice cultivars were also analyzed. Overall, our findings will provide a foundation for exploring the molecular mechanisms of alkali-resistant genes.

## Results

### Sequencing statistics

A total of 36.04 million, 40.16 million, 30.49 million and 44.12 million raw reads were obtained from the Caidao and WD20342 transcriptome libraries under the normal condition (control) (marked as CD and WD) and alkaline treatment (marked as CDT and WDT), respectively (Table 1). More than 91.43% high-quality reads (clean reads) were obtained and used for downstream analyses (Table 1 and Supplementary Table S1). The alignment results showed that 78.83–88.32% of clean reads from all twelve samples could be mapped on the reference genome (Supplementary Table S2). On average, approximately 30.44 (84.86%) and 29.46 (83.28%) million reads were uniquely mapped on the reference genome with TopHat for Caidao and WD20342, respectively. The assembly of mapped reads resulted in the identification of a total of 57264 genes and 1463 novel transcripts in all of the samples (Table 1). Unlike WD, the number of genes expressed in CD is significantly reduced under alkaline stress (more than 4400) (Table 1). A total of 5186 genes were expressed in CD without expression in CDT (Supplementary Table S3). This result showed that alkaline stress had a greater effect on CD than WD.

**Table 1:** Statistics of transcriptome sequencing results.

Sample	CD	CDT	WD	WDT
Raw reads	36040211	40164069	30489572	44115364
Clean reads	34151831	38468190	29004291	42498490
Total mapped	28376841 (83.09%)	33323238 (86.63%)	23726722 (81.80%)	36027328(84.77%)
Uniquely mapped	27939733 (81.81%)	32931934 (85.61)	23366350(80.56)	35567242 (83.69%)
Multiple mapped	437107 (1.28%)	391304 (1.02%)	360373 (1.24%)	460086 (1.08%)
Expressed gene	21680	17220	19642	18993
Novel transcripts	764	430	658	592

### Identification of DEGs

**Figure 1:**
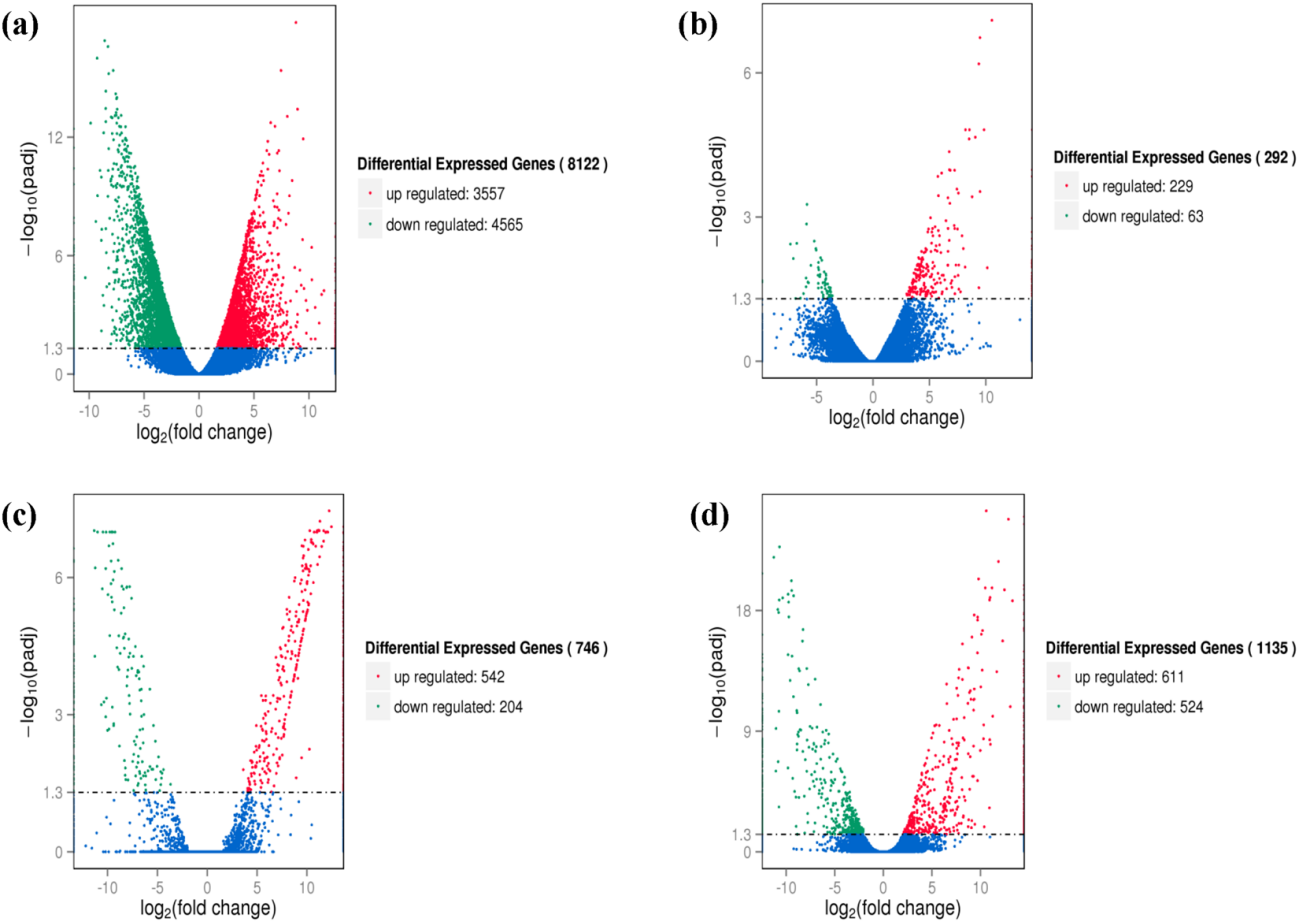
Volcano plots for expressed genes in the four comparison groups. Volcano plots for all the expressed genes in (**a**) CDT vs. CD, (**b**) WDT vs. WD, (**c**) CD vs. WD, and (**d**) CDT vs. WDT. X- and Y-axis present the *log*_2_(*ratio*) for the two samples and −*log*10(padj), respectively. Red (Up regulated) and green (down regulated) dots mean that the genes have significant difference, while the blue dots correspond to genes with no significant differences.

By comparing samples of the same rice cultivar in different conditions (control and stress) and different rice cultivars (Caidao and WD20342) in the same condition, we constructed four comparison groups: CDT vs. CD, WDT vs. WD, CD vs. WD and CDT vs.WDT. The volcano plots of the DEGs for the four groups are shown in Fig. 1. By restricting −log_10_ (Padj) > 1.3 (Padj < 0.05), the analysis revealed a total of 8122, 292, 746, and 1135 significant DEGs in CDT vs. CD, WDT vs. WD, CD vs. WD and CDT vs.WDT, respectively. The results indicated that significant differences in the level of gene expression occurred within not only the cultivars but also the treatments. In the comparison groups with two different cultivars, 611 DEGs were up-regulated and 524 DEGs were down-regulated among the 1135 DEGs in CDT vs.WDT and there were 542 and 204 DEGs that were up-regulated or down-regulated among the 746 DEGs in CD vs. WD. The comparison between WDT vs. WD and CDT vs. CD revealed 229 up-regulated and 63 down-regulated DEGs in WDT vs. WD compared with 3557 up-regulated and 4565 down-regulated DEGs in CDT vs. CD. It was obvious that more DEGs were up-regulated in WDT vs. WD after alkaline stress, however, more DEGs were down-regulated in CDT vs. CD. Furthermore, more DEGs in the alkali-sensitive cultivar were alkaline stress responsive compared with the alkali-resistant cultivar under alkaline conditions (Fig. 1). This result indicated that alkaline stress had a greater effect on the alkali-sensitive cultivar than on the alkali-resistant cultivar. Additionally, a total of 120 differentially expressed novel transcripts were identified in the four comparison groups.

### Classification of DEGs

**Figure 2:**
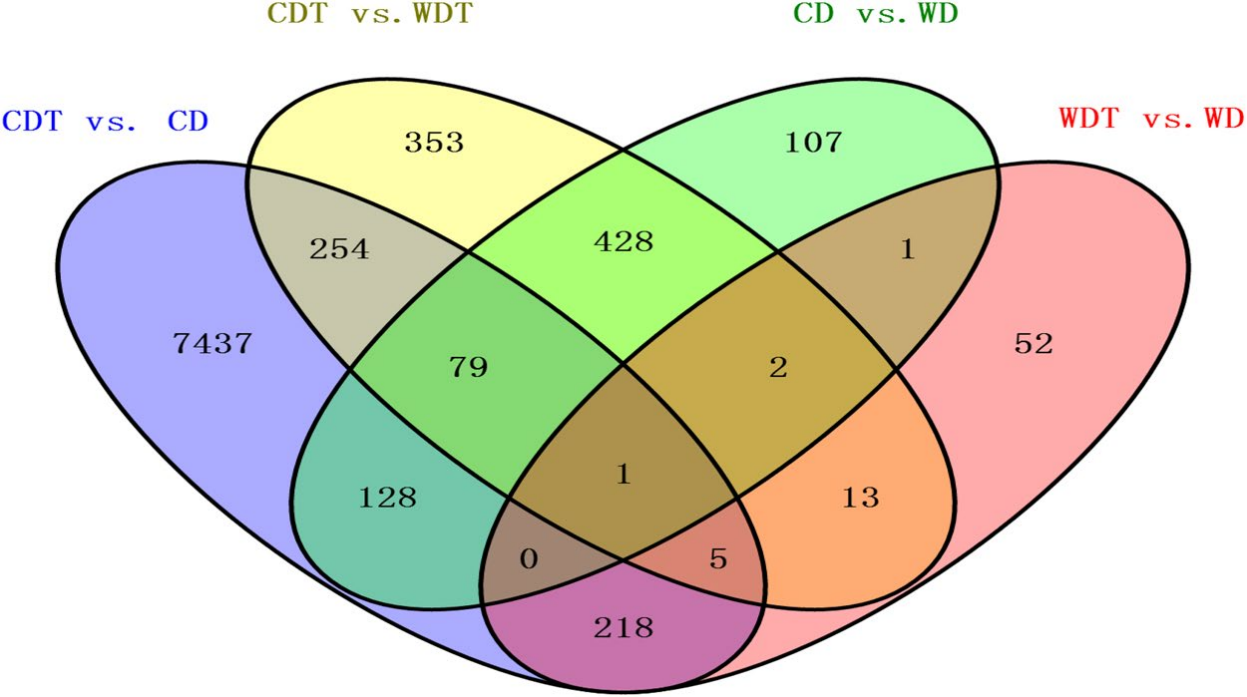
Venn diagrams for DEGs in the four comparison groups.

A total of 9078 unique DEGs were identified in all four groups. These DEGs could be divided into 15 disjointed subgroups, among which 81.92% (7437/9078), 0.57% (52/9078), 1.18% (107/9078), and 3.89% (353/9078) were group-specific DEGs in CDT vs. CD, WDT vs. WD, CD vs. WD and CDT vs.WDT, respectively. The Venn diagram of the 9078 unique DEGs in the four groups is shown in Fig. 2. Only one DEG (*LOC_Os06g51050*) was commonly expressed across all four groups.

Among the 15 sets of DEGs, the eight groups that contained CD vs. WD were excluded from the downstream analysis because CD and WD were not treated by alkaline stress. The seven remained subgroups were further classified into three categories: genes from the sensitive cultivar with alkali-responsive (SAR), genes from the resistant cultivar with alkali-responsive (RAR), and common (non cultivar-specific) alkali-responsive (CAR) DEGs. These three categories contained 7691, 65 and 576 DEGs, respectively, composing 84.72%, 0.72% and 6.35% of the 9078 DEGs, respectively. A total of 3, 301 and 32 novel transcripts were detected in the RAR, SAR and CAR, respectively (Supplementary Table S4). Detailed conditions for the classifying are shown in Table 2.

**Table 2:** Classification of three categories of DEGs.

Categories	Subgroups	Number of DEGs
SAR	Only CDT VS.CD	7437
	CDT VS.CD, CDT VS.WDT	254
RAR	Only WDT VS.WD	52
	WDT VS.WD, CDT VS.WDT	13
CAR	Only CDT VS.WDT	353
	CDT VS.CD, WDT VS.WD	218
	CDT VS.CD, WDT VS.WD, CDT VS.WDT	5

Subsequently, we primarily analyzed the above three categories of DEGs and screened important alkali-responsive (IAR) genes. Firstly, based on the expression levels of the three categories of DEGs, we performed a second round of DEG selection. There were 962 IAR genes screened by restricting padj < 0.01 and |log_2_ (*Ratio*) | å 5. Supplementary Table S5 shows detailed information on the 962 IAR genes in the final selection. Among the 962 IAR genes, 28 were RAR, 771 were SAR and 163 were CAR.

### GO enrichment analysis

**Figure 3:**
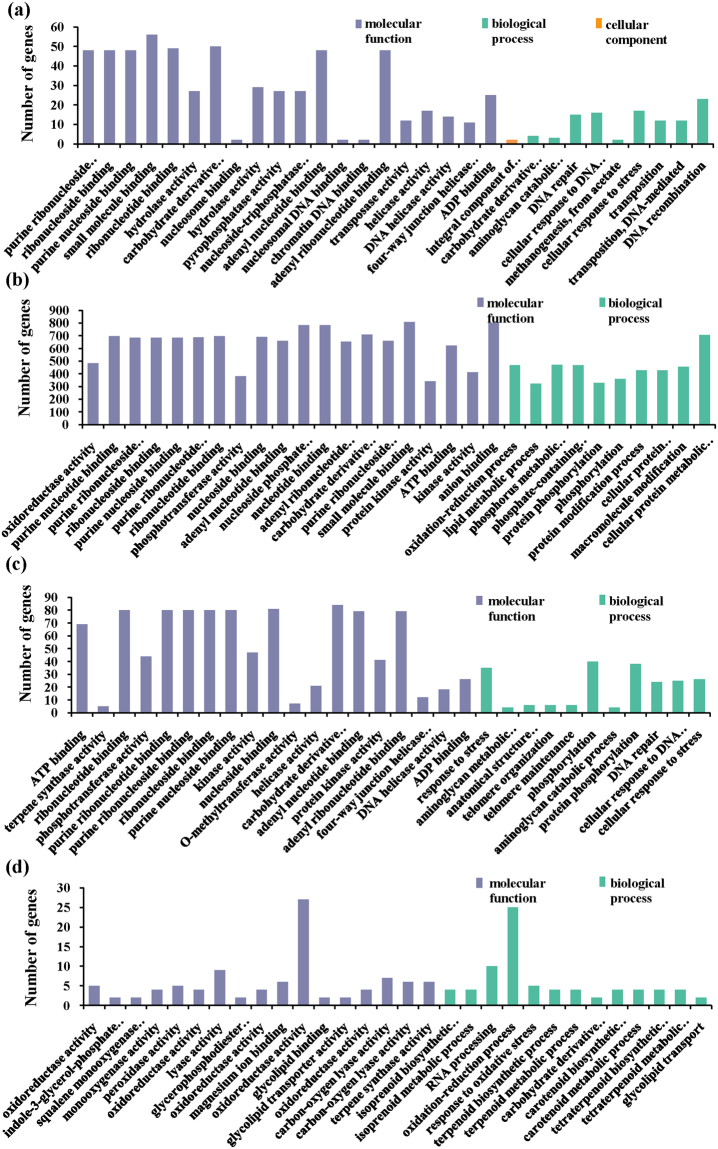
The most significantly-enriched GO terms of DEGs from the four comparison groups. (**a**) CD vs. WD, (**b**) CDT vs. CD, (**c**) CDT vs. WDT, (**d**) WDT vs. WD.

For all DEGs in the four comparison groups, a total of 4231(52.09%), 163(55.82%), 283(37.94%) and 471(41.50%) DEGs were assigned GO terms in CDT vs. CD, WDT vs. WD, CD vs. WD and CDT vs. WDT, respectively. The cellular protein metabolic process was the most significantly represented group in the biological process category, suggesting that extensive metabolic activities were occurring in the rice seedlings with alkaline treatment. Within the cellular component category, integral component of plasma membrane was the most significantly represented group, and anion binding was the most significantly represented group within the molecular functional category (Fig. 3).

**Figure 4:**
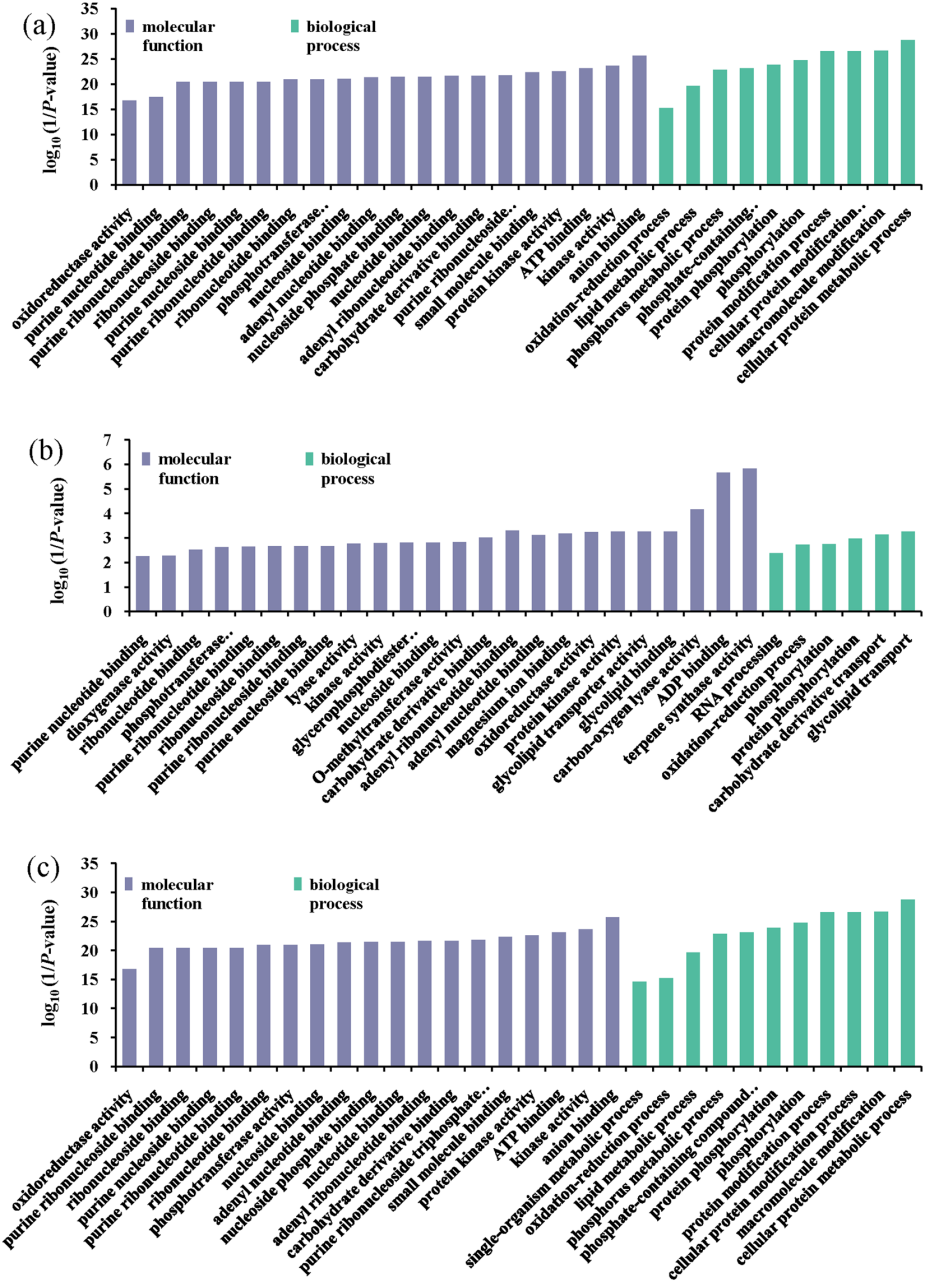
The most significantly-enriched GO terms of DEGs from SAR, RAR and CAR. (**a**) SAR, (**b**) RAR, (**c**) CAR.

We further identified GO terms categories that were over-represented (*P*-value < 0.05) in DEGs of RAR, SAR and CAR (Fig. 4, Supplementary Table S6). Among the 28 DEGs that were RAR, 19 were involved in 86 different GO terms among which ten and eight DEGs were involved in various enzyme activities and metabolic processes, respectively (Fig. 4b). Among the ten DEGs involved in the response to enzyme activity, seven were up-regulated in WDT compared with WD. Moreover, more than five genes were involved in oxidoreductase activity. Two DEGs and one DEG were involved in terpene synthase and dioxygenase activity, respectively.

For the selected 771 DEGs that were SAR, 481 had GO annotations and were involved in 429 different GO terms (Fig. 4a). Among 456 DEGs, 36 genes were involved in metabolic processes, such as cellular protein metabolic process, phosphate-containing compound metabolic process, lipid metabolic process and carbohydrate derivative metabolic process. Nineteen DEGs that were SAR also enriched in biosynthetic processes, such as lipid biosynthetic process, amide biosynthetic process and carbohydrate biosynthetic process. For the selected 163 DEGs that were CAR, 84 were involved in 278 different GO terms (Fig. 4c). Nineteen DEGs in CAR were involved in various enzyme activities, such as kinase activity, phosphotransferase activity and oxidoreductase activity.

### KEGG pathway enrichment analysis

To further investigate the DEGs that were involved and enriched in various metabolic pathways, pathway-based analysis was performed using the KEGG pathway database. In this analysis, 86 of 746 DEGs in the CD vs. WD, 2923 of 8122 DEGs in the CDT vs. CD, 150 of 1135 DEGs in the CDT vs. WDT and 115 of 292 DEGs in the WDT vs. WD were classified into 42, 119, 56 and 50 functional categories, respectively. We further identified over-represented KEGG Orthology (KO) terms (*P*-value < 0.05) and classified these terms into 26 categories (Table 3).

**Table 3:** Analysis of KEGG enrichment for DEGs from the four comparison groups.

KEGG pathway	Number of genes	*P*-value
**CD VS. WD**		
Peroxisome	4	0.00824
Citrate cycle (TCA cycle)	3	0.014718
Glyoxylate and dicarboxylate metabolism	3	0.021394
**CDT VS. CD**		
Ribosome	148	0.001675
Tryptophan metabolism	14	0.006377
Fatty acid degradation	26	0.008806
Plant hormone signal transduction	84	0.011027
Limonene and pinene degradation	12	0.015622
Porphyrin and chlorophyll metabolism	22	0.016304
alpha-Linolenic acid metabolism	21	0.017051
Phenylalanine, tyrosine and tryptophan biosynthesis	24	0.026828
Biosynthesis of secondary metabolites	296	0.027286
Valine, leucine and isoleucine degradation	21	0.031889
Glycerolipid metabolism	24	0.032142
Fatty acid metabolism	36	0.03252
Cutin, suberine and wax biosynthesis	12	0.039417
**CDT VS. WDT**		
Diterpenoid biosynthesis	5	0.000178
Biosynthesis of secondary metabolites	23	0.007861
Fatty acid elongation	3	0.010221
Limonene and pinene degradation	2	0.029038
Cutin, suberine and wax biosynthesis	2	0.040045
Stilbenoid, diarylheptanoid and gingerol biosynthesis	2	0.044012
**WDT VS. WD**		
Ribosome biogenesis in eukaryotes	9	4.97E-06
Plant hormone signal transduction	6	0.036989
Galactose metabolism	3	0.037562
Starch and sucrose metabolism	5	0.045365

For the selected 28 DEGs that were RAR, only one, *LOC_Os03g64260*, was involved in one over-represented pathway, the plant hormone signal transduction pathway (Table 4). Among the DEGs that were SAR, 72 of the 771 were involved in 15 different over-represented pathways among which nine were involved in plant hormone signal transduction pathways, 34 were involved in the biosynthesis of secondary metabolites pathways (Table 4). Among the 163 DEGs that were CAR, only six were involved in three different over-represented pathways, with one gene involved in the plant hormone signal transduction pathway, one gene involved in the alpha-Linolenic acid metabolism pathway and four genes involved in the biosynthesis of secondary metabolites pathways (Table 4). This result suggested that plant hormone signal transduction pathway and biosynthesis of secondary metabolites pathways might have a modulating effect on the regulation of alkali-responsive gene expression. These annotations will provide a valuable resource for investigating associated pathways of alkali-stress responses in rice.

**Table 4:** Analysis of KEGG enrichment for IAR DEGs from the three categories.

KEGG pathway	Number of genes	*P*-value
**RAR**		
Plant hormone signal transduction	1	0.036989
**SAR**		
Ribosome	6	0.001675
Tryptophan metabolism	1	0.006377
Fatty acid degradation	3	0.008806
Plant hormone signal transduction	9	0.011027
Porphyrin and chlorophyll metabolism	2	0.016304
alpha-Linolenic acid metabolism	2	0.017051
Phenylalanine, tyrosine and tryptophan biosynthesis	1	0.026828
Biosynthesis of secondary metabolites	34	0.027286
Valine, leucine and isoleucine degradation	1	0.031889
Glycerolipid metabolism	1	0.032142
Fatty acid metabolism	2	0.032520
Cutin, suberine and wax biosynthesis	6	0.039417
Limonene and pinene degradation	1	0.029038
Stilbenoid, diarylheptanoid and gingerol biosynthesis	1	0.044012
Diterpenoid biosynthesis	2	0.000178
**CAR**		
Biosynthesis of secondary metabolites	4	0.027285
Plant hormone signal transduction	1	0.011027
alpha-Linolenic acid metabolism	1	0.017051

### Identification of differentially expressed transcription factors

**Figure 5:**
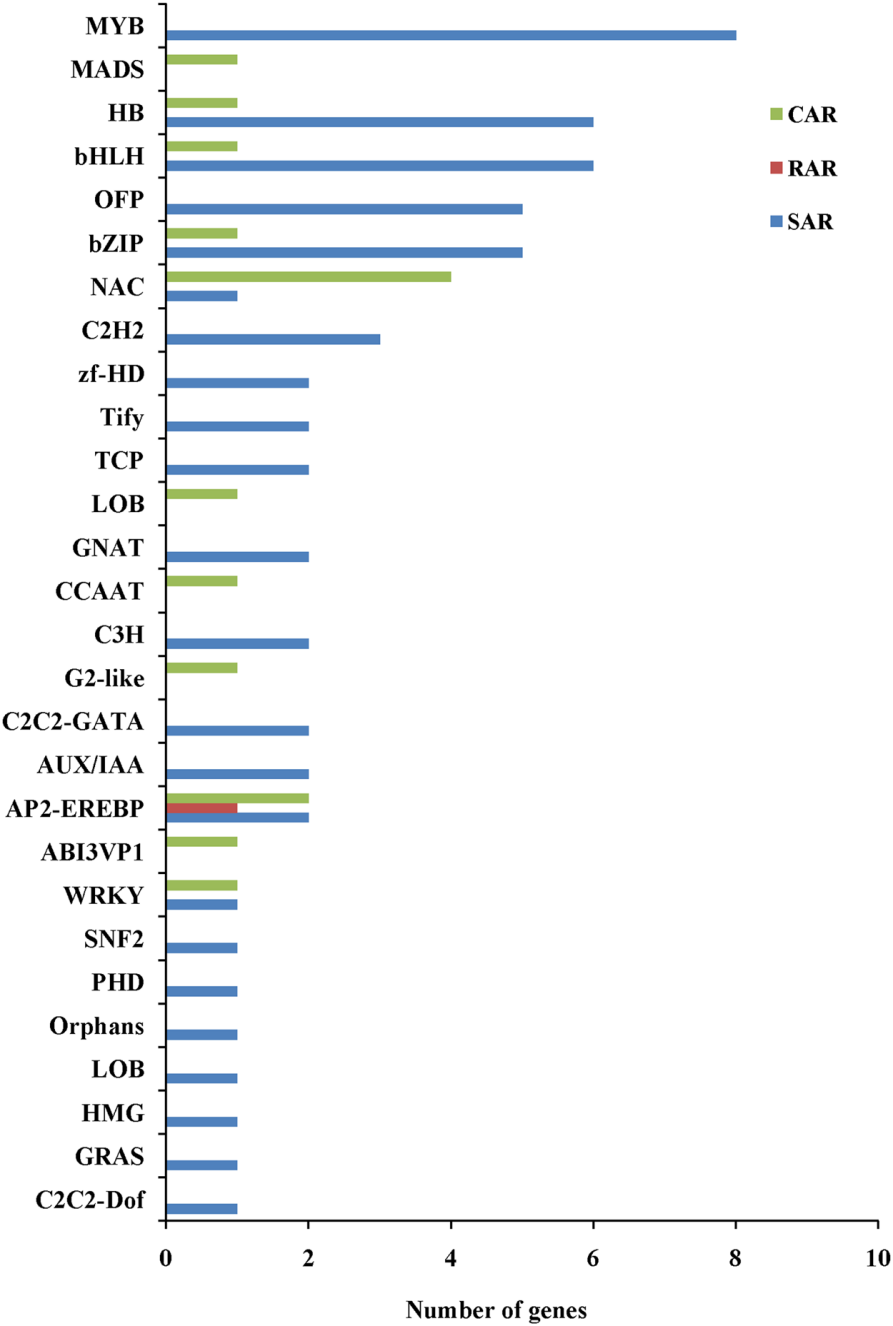
Transcription factor families of the DEGs in CAR, RAR and SAR.

Transcription factors (TFs) play critical roles in responding to various types of abiotic stress^29^. In this study, we analyzed differential expression of TFs in CDT vs. CD, WDT vs. WD, CD vs. WD and CDT vs.WDT. A total of 576 TFs were differentially expressed in all four groups. These TFs were included in 69 TF families, such as MYB (47), WRKY (40), NAC (39), AP2-EREBP (35), bHLH (31), and bZIP (30) (Supplementary Fig. S2). Among these 576 TFs, one was RAR, 58 were SAR and 15 were CAR (Fig. 5). Some of these TFs were involved in responses to various abiotic stresses according to previous studies. In future investigations, whether other TFs also play important roles in the alkaline stress response and in plant tolerance to stress challenges in general will be important to determine.

### Differential alternative splicing analysis

**Figure 6:**
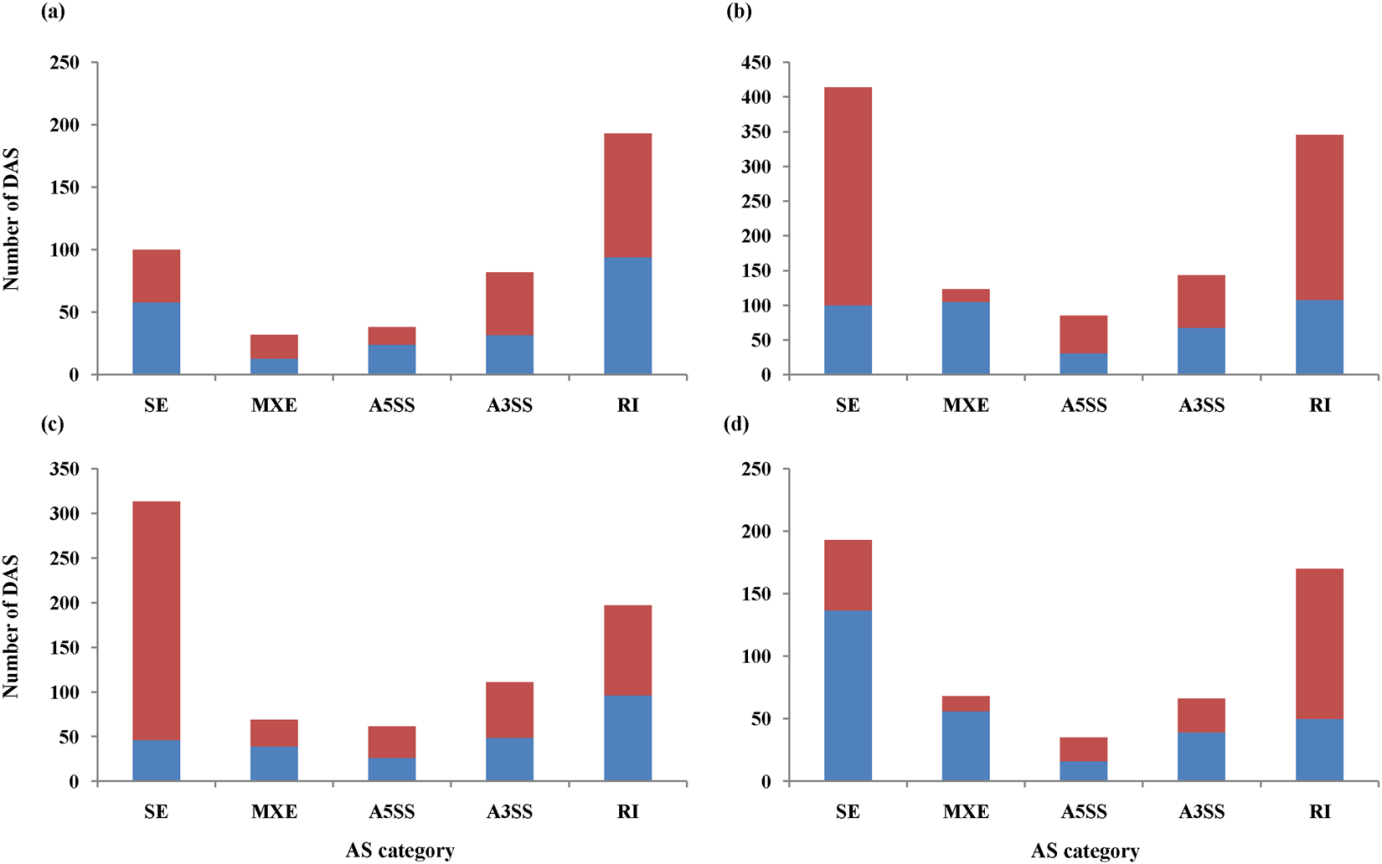
Differential alternative splicing (DAS) events in the four comparison groups. (**a**) CD vs. WD, (**b**) CDT vs. CD, (**c**) CDT vs. WDT, (**d**) WDT vs. WD. The blue bars represent up-regulated DAS events and the red bars represent down-regulated DAS events.

Alternative splicing (AS) is an important mechanism in the regulation of eukaryotic genes^30^. We defined five primary classes of AS events: retained intron (RI), skipping exon (SE), alternative 5′ splice site (A5SS), alternative 3′ splice site (A3SS), and the mutually exclusive exon (MXE). In this study, we detected differential alternative splicing (DAS) in the four comparison groups. In total, 2838 (1110, 532, 445 and 751 DAS in CDT vs. CD, WDT vs. WD, CD vs. WD and CDT vs.WDT, respectively) DAS that distributed across 1512 genes were identified (Fig. 6) (FDR < 0.05). SE and RI were the most predominant DAS types (>65%) in the four comparison groups.

For the selected 771 DEGs that were SAR, nine occurred with DAS in CDT vs. CD or CDT vs.WDT. *LOC_Os04g52500* and *LOC_Os02g54254* occurred simultaneously with MXE and SE in CDT vs. CD. *LOC_Os06g13560* occurred with RI both in CDT vs. CD and CDT vs.WDT. The remaining six genes occurred with either MXE or SE in CDT vs. CD or CDT vs.WDT (Table 5).

**Table 5:** Differential alternative splicing analysis for IAR DEGs from the three categories.

Event type	Groups	Gene	FDR
**SAR**			
MXE	CDT VS. CD	*LOC_Os04g52500*	1.95E-25
		*LOC_Os05g11560*	4.51E-11
		*LOC_Os02g54254*	7.72E-24
	CDT VS. WDT	*LOC_Os02g09810*	1.86E-10
		*LOC_Os05g39770*	2.53E-42
RI	CDT VS. CD	*LOC_Os06g13560*	1.55E-09
	CDT VS. WDT	*LOC_Os06g13560*	0.005939
SE	CDT VS. CD	*LOC_Os04g52500*	3.87E-07
		*LOC_Os02g54254*	1.11E-19
	CDT VS. WDT	*LOC_Os03g49380*	7.09E-44
		*LOC_Os04g10060*	0.000605
		*LOC_Os02g02210*	0.043366
**CAR**			
A3SS	CDT VS. CD	*LOC_Os05g46460*	1.09E-06
	CDT VS. WDT	*LOC_Os05g46460*	7.49E-08
MXE	CDT VS. WDT	*LOC_Os08g30020*	0.011291
		*LOC_Os12g26290*	3.87E-64
	WDT VS. WD	*LOC_Os05g46460*	0.002765
		*LOC_Os08g30020*	0.000242
		*LOC_Os12g26290*	5.73E-46
RI	CDT VS. CD	*LOC_Os05g46460*	1.14E-16
	CDT VS. WDT	*LOC_Os05g46460*	1.83E-05
	WDT VS. WD	*LOC_Os07g38840*	7.74E-08
SE	CDT VS. WDT	*LOC_Os03g55660*	1.99E-07
		*LOC_Os05g33570*	6.07E-05
	WDT VS. WD	*LOC_Os12g26290*	0.008074

Among the 163 DEGs that were CAR, six occurred with DAS. *LOC_Os05g46460* occurred simultaneously with A3SS and RI in CDT vs. CD and CDT vs. WDT. *LOC_Os08g30020* and *LOC_Os12g26290* occurred with MXE both in WDT vs. WD and CDT vs.WDT. Furthermore, *LOC_Os12g26290* also occurred simultaneously with MXE and SE in WDT vs. WD (Table 5).

### qRT-PCR validation

**Figure 7:**
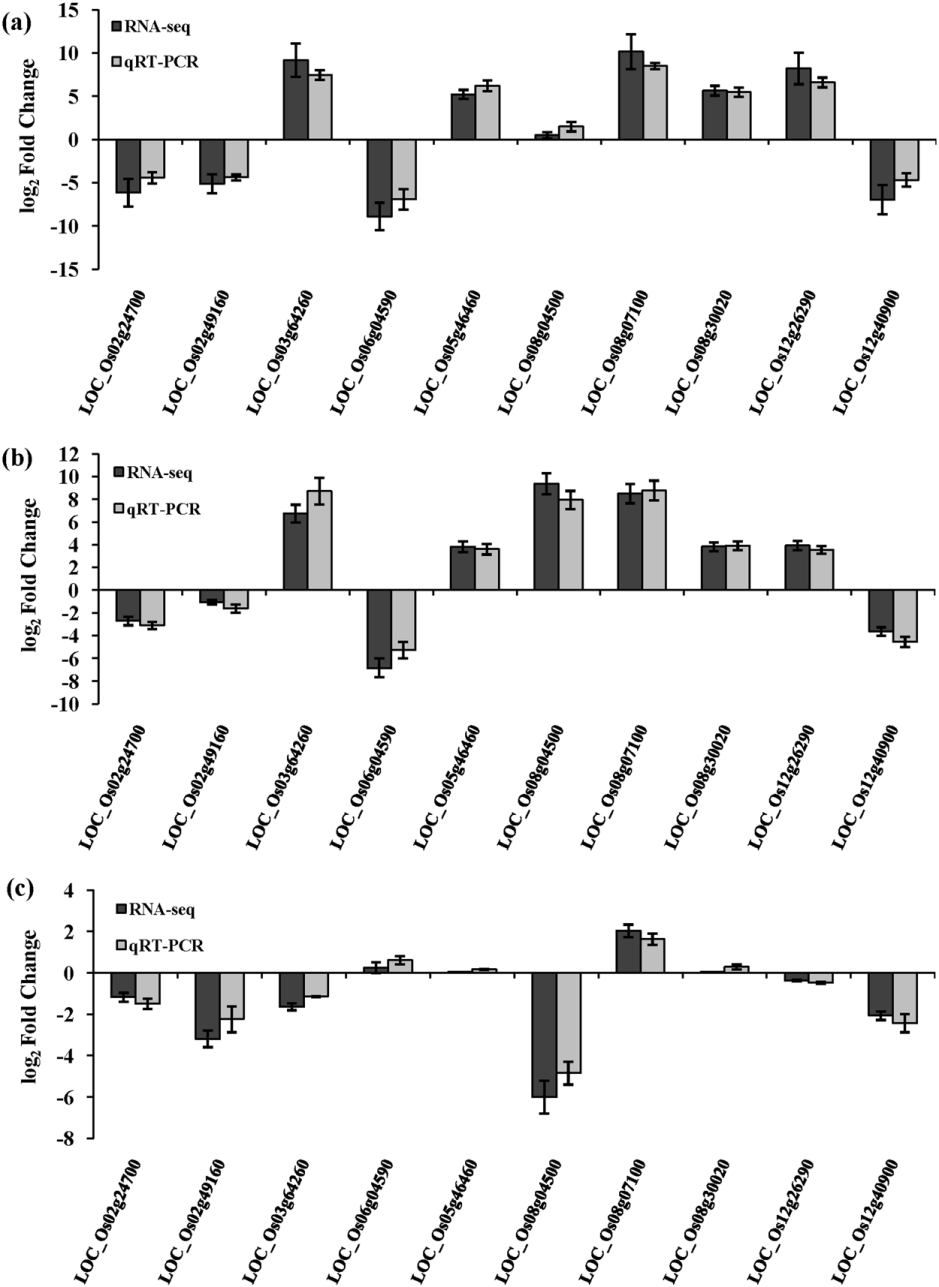
Comparison of RNA-seq results and qRT-PCR analysis of gene expression levels. (**a**) Log_2_ fold change of 10 genes in CDT vs. CD. (**b**) Log_2_ fold change of 10 genes in WDT vs. WD. (**c**) Log_2_ fold change of 10 genes in CDT vs. WDT.

To confirm the accuracy and reproducibility of the Illumina RNA-Seq results, ten representative genes were chosen to validate the levels of expression before and after the alkaline treatment by quantitative real-time PCR (qRT-PCR). The validation results for the ten genes are shown in Fig. 7. Four genes, *LOC_Os02g49160*, *LOC_Os02g24700*, *LOC_Os06g04590* and *LOC_Os12g40900*, were SAR, three genes, *LOC_Os03g64260*, *LOC_Os08g07100*, and *LOC_Os08g04500*, were RAR and the final three genes, *LOC_Os05g46460*, *LOC_Os08g30020* and *LOC_Os12g26290*, were CAR. Among these ten genes, the four genes that belonged to SAR were all involved in the auxin signal transduction pathway. *LOC_Os03g64260* was involved in the ethylene signal transduction pathway, *LOC_Os08g04500* and *LOC_Os08g07100* were involved in terpene synthase activity. The final three genes that were CAR all occurred with DAS in the comparison groups. Based on the RNA-seq results, the four genes that were SAR were all down-regulated in expression in CDT vs. CD. *LOC_Os03g64260*, *LOC_Os08g04500* and *LOC_Os08g07100* were all up-regulated in WDT compared with WD. Moreover, *LOC_Os05g46460* and *LOC_Os08g30020* were up-regulated in CDT vs. CD and WDT vs. WD, simultaneously. The relative trends in the expression patterns of the qRT-PCR results were all consistent with the RNA-Seq data, although some differences were detected in the absolute expression levels (Fig. 7).

## Discussion

Alkalinity is a highly stressful environmental factor that limits plant growth and production^31–33^. Alkaline stress and salt stress are often interconnected and may induce mixed effects, such as osmotic, specific ion and high-pH effects, and therefore are difficult to control and engineer^34–36^. Studies have used stress-responsive genes to improve the resistance of rice to adverse environments by gene transformation^37–39^, and many genes are involved in the responses of rice to various abiotic stresses^40–43^. Studies in recent years have provided valuable information on the molecular mechanisms for stress resistance in plants based on morphological, physiological and molecular responses.

It has been reported, more genes were responded in leaves than roots under stress treatment^25,27^. According to our observation, alkaline stress-sensitive (WD20342) and stress-tolerant rice (Caidao) cultivar showed obvious phenotypic difference in leaves when using 0.5% Na_2_CO_3_ treatment after 36 h, but only showed subtle difference roots after 36 h. Therefore, in order to obtain gain a deeper understanding of the gene level variations between the alkaline stress-sensitive and stress-tolerant rice cultivars, we performed transcriptomic analyses of leaves of two rice varieties (WD20342 and Caidao) under two experimental conditions (control and alkaline). The statistical analysis revealed a total of 9078 unique DEGs in four comparison groups. By further selecting DEGs with large differences and low padj in expression levels among paired samples, 962 DEGs were ultimately selected as important alkali-responsive (IAR) genes. Among these DEGs, we found that some of the genes were involved in responding to salt stress or improving tolerance to high-salinity stress, according to previous studies. For example, *LOC_Os11g45740* was differentially expressed between CDT and CD, with log_2_ (CDT/CD) = 5.1017. In fact, *LOC_Os11g45740* is a rice R2R3-type MYB transcription factor gene, *Jamyb*, overexpressing in transgenic *Arabidopsis* that improves tolerance to high-salinity stress during seed germination^44^. Kothari *et al*.^45^ found that *OsAMTR1* (*LOC_Os05g39770*) was stress-responsive and showed increased expression under salt stress treatments. In our study, some of the genes related to rice responses to salt stress, such as *OsHAK21* (*LOC_Os03g37930*)^46^, *OsCYL2* (*LOC_Os06g43180*)^47^ and *Oshox12* (*LOC_Os03g10210*)^48^, also had very high absolute log_2_ fold-change values between the two samples in our experiment. Among the above five genes, *LOC_Os03g10210*, which was up-regulated by 6.7045 and 4.4174 fold in CDT vs. CD and WDT vs. WD, respectively. *LOC_Os03g37930* was up-regulated by 6.7620 and 6.3568 fold in CDT vs. CD and WDT vs. WD, respectively. *LOC_Os05g39770* was up-regulated by 5.8191 and 2.6475 fold in CDT vs. CD and WDT vs. WD, respectively. *LOC_Os06g43180* was down-regulated by 5.3941 and 2.4419 fold in CDT vs. CD and WDT vs. WD, respectively. *LOC_Os11g45740* was up-regulated by 5.1017 and 0.7044 fold in CDT vs. CD and WDT vs. WD, respectively. However, some salt stress-responding genes of rice were not included in the 962 IAR DEGs. We offer two potential explanations for the inconsistencies between our results and those of previous studies. Firstly, two different stress conditions were imposed in the studies. The previous studies were conducted under conditions of salt (NaCl) stress, whereas our study was based on RNA-seq of four samples under conditions of alkaline (Na_2_CO_3_) stress. Secondly, another possible explanation was that many salt stress-responding genes of rice had very low absolute log_2_ fold-change values in our experiment and therefore were excluded from the subsequent analyses. Further verification of the alkaline response of the identified genes in the two stress conditions using a combination of experimental designs would be of value.

In addition, we found that some of the DEGs were involved in ion transporter. Solute transport system is one of the major ways in which organisms interact with the environment. Transport is controlled by integral membrane proteins, of which one of the largest groups is the ATP-bind cassette (ABC) transporter protein^49^. Five and two ABC transporter genes were up-regulated and down-regulated in CDT vs. CD, respectively. Two *ALMT* genes were differentially expressed between CDT and CD (Table 6). The ALMT gene family has been considered as an important mechanism for plant resistance to abiotic stress^49^. A gene (*LOC_Os04g49570*) encoding GLR was down-regulated in CDT vs. CD after alkaline stress (Table 6). GLR was involved in many biological processes including light signaling, root-tip meristematic cell activity, pollen tube growth, cytosolic calcium ion flux and response to varied biotic and abiotic stresses^50^. Three *NRT* genes differentially expressed between CDT and CD (Table 6), which encode the NRTs and might be involved in the transfer of nitrate^51^. Among them, *LOC_Os10g40600* also differentially expressed between WDT and WD. *OsNRT2.3b* is located on the plasma membrane, expresses mainly in the phloem, and has a regulatory motif on the cytosolic side that acts to switch nitrate transport activity on or off by a pH-sensing mechanism.

**Table 6:** List of transport-related genes among the DEGs detected by RNA-Seq.

Gene ID (name)	Gene annotation/Function	FDR	Log_2_FC
*LOC_Os12g22110*	ABC-2 type transporter	0.00334	−3.0193
*LOC_Os11g39020*	ABC transporter	0.007371	3.9748
*LOC_Os11g05700*	ABC transporter	0.023961	2.8287
*LOC_Os09g39910*	ABC transporter	0.001744	3.0473
*LOC_Os08g43120*	ABC transporter	1.55E-07	9.4171
*LOC_Os08g30770*	ABC transporter	0.00254	3.3529
*LOC_Os04g49570* (*GLR3.1*)	Glutamate receptor (GLR)	0.005568	−2.4478
*LOC_Os10g42180*	Aluminum activated malate transporter (ALMT)	0.049301	1.7209
*LOC_Os06g15779*	Aluminum activated malate transporter (ALMT)	0.00494	−2.521
*LOC_Os10g40600* (*NRT1.1B*)	Nitrate transporter (NRT)	4.35E-12	−6.7887
*LOC_Os02g02190* (*OsNRT2.2*)	High-affinity Nitrate Transporter	0.011704	5.4363
*LOC_Os01g50820* (*OsNRT2.3*)	High-affinity Nitrate Transporter	0.019461	2.5345
*LOC_Os03g06410*(*OsMAPKKK1*)	Mitogen-activated protein kinase kinase kinase	0.003639	2.3836
*LOC_Os02g54600* (*OsMKK4*)	Mitogen-activated protein kinase (MAPK)	0.006223	2.6097
*LOC_Os09g36420* (*OsHSP50.2*)	Heat shock protein HSP)	0.012639	−2.5661
*LOC_Os08g39140* (*hsp82A*)	Heat shock protein (HSP)	0.009624	2.3063
*LOC_Os03g16860* (*OsHSP71.1*)	Rice heat shock protein gene	0.001602	3.0682
*LOC_Os12g41490*	Cysteine-rich receptor-like protein kinase (CPK)	8.61E-06	4.345
*LOC_Os11g44860*	Cysteine-rich receptor-like protein kinase (CRK)	0.039593	2.3565

Moreover, we compared the DEGs obtained in this study with different groups of gene that are associated with different abiotic stress. Heat shock proteins (HSPs) are a series of proteins which are significantly expressed in organisms when plants respond to environmental stress. The HSP of plants plays a key role in reliving the injury caused by heat stress and improving the thermo-tolerance^52^. Three HSP genes differentially expressed between CDT and CD (Table 6). Receptor-like kinases (RLKs) are protein kinases existing in plants, and play an essential role in many plant signal transduction pathways. Cysteine-rich receptor-like kinases (CRKs) are a large subgroup of RLKs and have been found to be involved in plant response to abiotic stresses^53^. Two CRK genes differentially expressed between CDT and CD (Table 6). The MAPK cascade is an important signaling module and plays a critical role in response to biotic and abiotic stresses as well as plant growth and development^54^. Two MAPK genes also genes differentially expressed between CDT and CD (Table 6).

GO enrichment analysis was used to reveal preferred GO terms and putative functional annotation for the DEGs. GO enrichment analysis revealed that the DEGs were enriched in responses to various stimuli or stresses, such as response to oxidative stress, metal ion, Gram-negative bacterium, drug, stimulus, chemical, stress, DNA damage stimulus and biotic stimulus. Similar to salt stress, alkaline stress is a typical abiotic stress^55^, and enrichment for the DEGs in the stimuli or stresses terms illustrated that the selected DEGs might be actual alkali-responsive DEGs. For example, the response to oxidative stress may contribute to the removal of active oxygen by increasing the activity of antioxidant enzymes^56,57^. Based on previous research, salt, drought, or cold stress causes an imbalance between the productions of reactive oxygen species (ROS) and antioxidant defenses. Such an imbalance results in oxidative stress, which causes toxic effects to all components of the cell, including proteins, lipids, and DNA, and therefore is one of the primary causes of plant damage caused by environmental stress^58–60^. In the present study, the response to the oxidative stress was enriched in CDT vs. CD, WDT vs. WD, and CDT vs. WDT but reduced in CD vs. WD. As we indicated, the DEGs in the comparison groups CDT vs. CD, WDT vs. WD, and CDT vs. WDT were all exposed to alkaline stress, but CD vs. WD was not treated. Moreover, two and two DEGs that enriched in ROS metabolic process were up-regulated and down-regulated in CDT vs. CD, respectively. One DEG that enriched in ROS biosynthetic process was up-regulated in CDT vs. CD. These five genes were only differentially expressed between CDT and CD. Therefore, the enrichment for related processes in the selected DEGs further supported their alkaline responsiveness.

KEGG enrichment analysis was performed to identify related pathways for the 962 IAR DEGs that were involved and enriched in this study. The plant hormone signal transduction pathway is one of the important pathways in plants^61,62^. Responses to adverse environmental conditions must be rapid and accurately coordinated to activate the necessary physiological changes that ensure plant growth and development, and these adaptive responses are usually mediated by plant hormones^63,64^. The primary plant hormones include auxin, cytokinin, gibberellin, ABA, ethylene, brassinosteroid, and jasmonic acid^65^.

Of the 962 IAR DEGs, ten were involved in plant hormone signal transduction pathways, including eight SAR (*LOC_Os02g49160*, *LOC_Os02g24700*, *LOC_Os04g56680*, *LOC_Os12g40900*, *LOC_Os04g32480*, *LOC_Os09g26780*, *LOC_Os06g04590* and *LOC_Os11g04600*), one RAR (*LOC_Os03g64260*) and one CAR (*LOC_Os01g64000*). Among these ten DEGs, *LOC_Os02g49160*, *LOC_Os02g24700*, *LOC_Os04g56680*, *LOC_Os12g40900* and *LOC_Os06g04590* were involved in the auxin signal transduction pathway, *LOC_Os04g32480* and *LOC_Os09g26780* were involved in the jasmonic acid signal transduction pathway, *LOC_Os11g04600* was involved in the salicylic acid signal transduction pathway, *LOC_Os01g64000* was involved in the abscisic acid signal transduction pathway, and the remaining one gene, *LOC_Os03g64260*, participated in the ethylene signal transduction pathway. The five genes involved in the auxin signal transduction pathway were all down-regulated in CDT compared with CD, and *LOC_Os06g04590* had the highest absolute CDT to CD fold-change ratio [log_2_ (CDT/CD) = −8.892]. For these five genes, the annotation functions were auxin-responsive small auxin-up RNA (SAUR) gene family member.

Auxin is an important plant hormone that is closely related with plant resistance to adverse environmental conditions^66^, Auxin can induce rapid and transient expression of some genes^67^, which primarily include auxin response factor genes (ARF) and primary auxin response genes (Aux/IAA, GH3, SAUR and LBD). In the current study, many members of auxin gene families were involved in rice responses to stress. For example, *OsIAA6* was induced in rice under high salt and drought condition^68^, and ABA and drought can improve transcription of GH3 to help maintain endogenous auxin at an appropriate level in rice^69^. Although the functions of Aux / IAA and GH3 family genes have been extensively studied, research on SAUR remains scarce.

**Figure 8:**
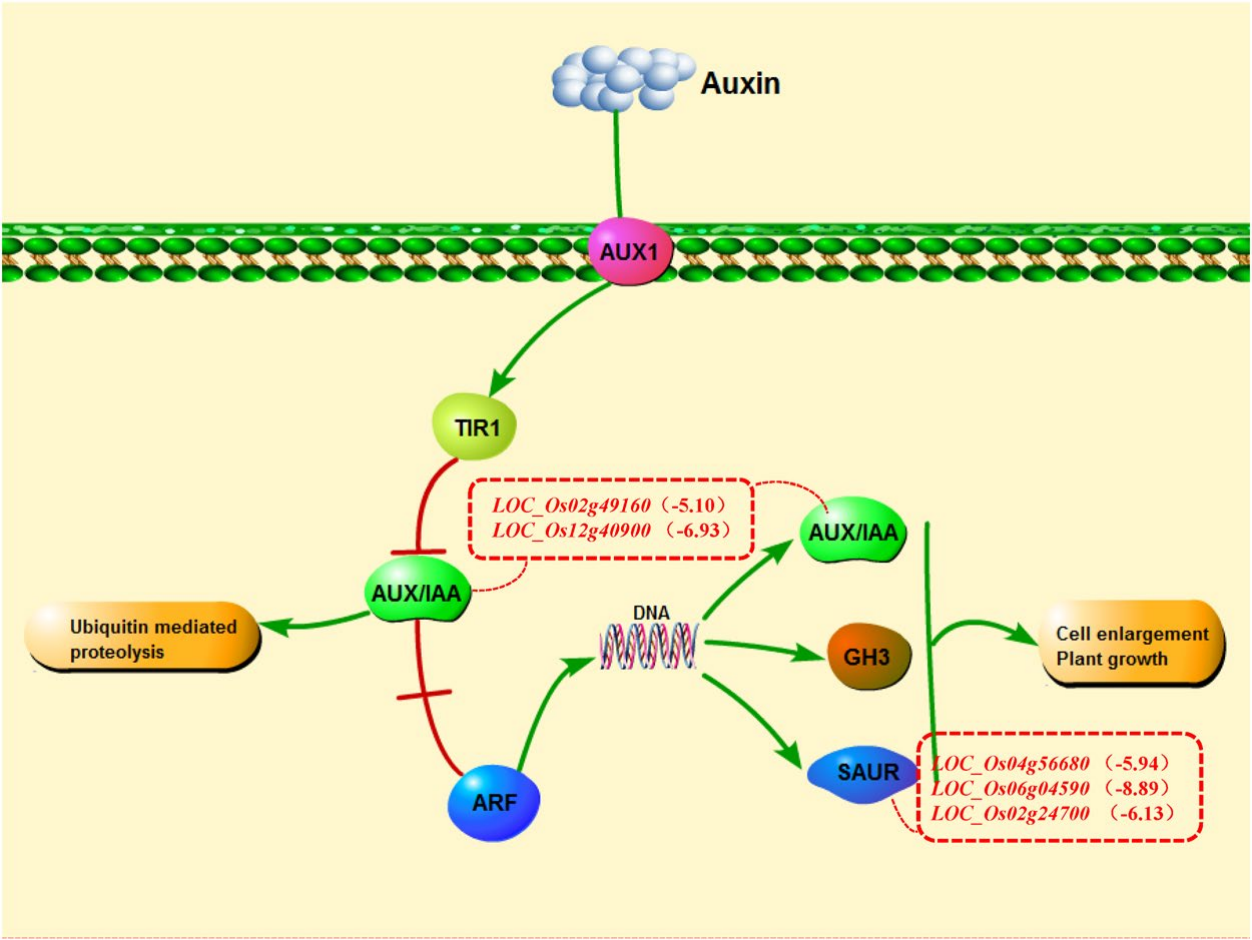
Five IAR genes involve in the plant hormone (Auxin) signal transduction pathway in KEGG.

The auxin-response signal transduction pathway is shown in Fig. 8. When the concentration of auxin increases, auxin combines with transport inhibitor response 1 (TIR1), causing Aux/IAA ubiquitination and degradation. Then, the auxin response factor (ARF) is released, which further activates the expression of SAUR genes. During this process, *LOC_Os02g49160* and *LOC_Os12g40900* encode an Aux/IAA gene, and *LOC_Os04g56680*, *LOC_Os06g04590* and *LOC_Os02g24700* encode a SAUR gene. Among these five genes, *LOC_Os12g40900* (*OsIAA3*) is one of the IAA gene family members whose expression increases rapidly in response to auxin^70^.

In plants, the phytohormone abscisic acid (ABA) plays a major role in the responses to a wide range of stresses, including drought, high salinity, and low temperature, and in developmental processes such as seed maturation, dormancy, and germination. AREB (ABA-responsive element binding protein)/ABF (ABRE binding factors) transcription factors can recognize and regulate the expression of ABA-responsive genes and enhance the ability of plant resistance to environmental stresses. One DEG, *LOC_Os01g64000*, that is related to the abscisic acid signal transduction pathway was found in only CAR. This DEG was up-regulated by 6.1496 and 3.5871 fold in CDT vs.CD and WDT vs. WD, respectively. In summary, analysis of plant hormone signal transduction pathways indicated that associated pathways were putatively alkali-responsive ones, and that the involved DEGs were actually related to many hormone signal transduction processes that respond to alkaline stress.

Transcription factors act as control switches in plant responses to abiotic stress responses^29^. Compared with previous studies, we also found that various TFs, such as MYB, WRKY, NAC, AP2-EREBP, bHLH, and bZIP, were enriched in WD20342 and Caidao under stress conditions^22,71,72^. Among the 962 IAR DEGs, we also found that 74 of these genes were TFs, including one RAR, 58 SAR and 15 CAR. Of these TFs, some demonstrated involvement in responding to various abiotic stresses in previous studies. For example, *OsJAMyb* (*LOC_Os11g45740*)^44^, *OsMYB2P-1*(*LOC_Os05g04820*)^73^, *ONAC131*(*LOC_Os12g03040*)^74^, *OsNAC10*(*LOC_Os11g03300*)^75^, *OsbZIP16*(*LOC_Os02g09830*)^76^ and *OsABI5*(*LOC_Os01g64000*)^77^ were induced by stress both in previous studies and in our results. This result suggested that TFs also play an important role in the alkaline stress response, and that the related TFs were actually involved in regulatory processes that affected tolerance to alkaline stress.

Recent studies have established that AS events are a crucial regulatory mechanism common in plants^30,78^. The AS events often lead to the production of multiple proteins in animals and plants, thereby enhancing biological diversity^79,80^. Notably, some proteins produced from specific AS transcripts were also related to salt stress responses in plants^79^. In the present study, we detected a difference in most of the AS events under alkaline stress in the four comparison groups. The change of the frequency of AS events may be a strategy employed by the rice toward energy conservation as a stress adaptive mechanism. The difference in the number of AS variants in Caidao and WD20342 under alkaline stress was an indication that the response mechanism to alkaline stress was different between the two cultivars. Therefore, genes associated with differential AS likely play an important role in adapting to alkaline stress. In this study, we found 15 genes of the 962 IAR DEGs that were associated with differential AS in CDT vs. CD, WDT vs. WD and CDT vs. WDT. These results implied that these genes play an important role in stress adaptation under alkaline stress conditions in rice.

## Conclusion

We provided a comprehensive overview of the transcriptome of two rice cultivars with the excavation of alkali-responsive DEGs, which highlighted the transcriptional variations among these DEGs under control and stress conditions. Statistical analysis of 9078 DEGs revealed three classes for a total of 962 IAR DEGs in rice. These important alkali-responsive DEGs were frequently involved in specific biological processes and metabolic pathways that might be important for alkaline stress tolerance in rice. Furthermore, for the selected 962 DEGs, 15 occurred with DAS and 74 were TFs. Overall, the IAR DEGs identified in this study can be used to identify most suitable candidate genes for future transgenic research with susceptible rice cultivars to generate high-yielding stress-tolerant rice cultivars.

## Methods

### Plant materials and alkaline stress treatment

Two rice cultivars with different levels of resistance to alkaline stress were studied: WD20342, which is resistant to alkaline stress, and Caidao, which is sensitive to alkaline stress. The seeds were surface sterilized with 2% sodium hypochlorite for 45 min and immersed in reverse osmosis (RO) water in the dark, and the uniformly germinated seeds were sown in 96-well plates supported by a plastic container containing Yoshida’s cultural solution^81^ in a culture room (14 h light/10 h dark at 28 ± 1 °C). When the seedlings reached the two-leaf stage, the seedlings of WD20342 and Caidao were subjected to control and alkaline stress treatment (marked as WDT and CDT, respectively). The seedlings of WD20342 and Caidao were kept grown on RO water served as controls (marked as WD and CD, respectively). For alkaline stress treatment, seedlings were transferred on their 96-well plates into containers filled with 0.5% Na_2_CO_3_ solution (pH = 11.37) for 36 h.

### RNA extraction

After alkaline stress, leaves of 5 plants from each treatment (control and alkaline) of each cultivar (WD20342 and Caidao) were harvested and pooled and was immediately frozen in liquid nitrogen. Three biological replicates were applied for each cultivar. Total RNA was extracted with TRIzol according to the manufacturer’s instructions (Invitrogen, USA). RNA degradation and contamination were monitored on 1% agarose gels. Total RNA was treated with DNase to remove all traces of DNA. RNA purity was checked using a NanoPhotometer^®^ spectrophotometer (IMPLEN, CA, USA). RNA concentration was measured using the Qubit^®^ RNA Assay Kit in a Qubit® 2.0 Flurometer (Life Technologies, CA, USA). RNA integrity was assessed using an RNA Nano 6000 Assay Kit of the Bioanalyzer 2100 system (Agilent Technologies, CA, USA).

### Transcriptome sequencing, quality control and mapping

A total of 3 µg of RNA per sample was used as input material for the RNA sample preparations. Sequencing libraries were generated using a NEBNext® Ultra™ RNA Library Prep Kit for Illumina® (NEB, USA) following the manufacturer’s recommendations and index codes were added to attribute sequences to each sample. First strand cDNA was synthesized using random hexamer primer and M-MuLV Reverse Transcriptase (RNase H^-^). PCR products were purified (AMPure XP system) and library quality was assessed on an Agilent Bioanalyzer 2100 system. The library was sequenced using the Illumina HiSeq. 2000 platform. A total of 12 samples were sequenced.

Clean data (clean reads) were obtained by removing reads containing adapters, reads containing poly-N and low quality reads from raw data. Simultaneously, Q20, Q30 and GC content of the clean data were calculated. All downstream analyses were based on the clean data with high-quality reads. Reference genome and gene model annotation files were downloaded from the genome website directly. Index of the reference genome was built using Bowtie v2.2.3, and paired-end clean reads were aligned to the reference genome using TopHat v2.0.12.

### Quantification of gene expression level

HTSeq v0.6.1 was used to count the number of reads mapped to each gene. Then, the FPKM of each gene was calculated based on the length of the gene and reads count mapped to that gene. FPKM, expected number of Fragments Per Kilobase of transcript sequence per millions base pairs sequenced, simultaneously considers the effect of sequencing depth and gene length for the reads count and is currently the most commonly used method for estimating gene expression levels^82^.

### Differential expression analysis

Differential expression analysis of two samples was performed using the DESeq R package (1.18.0). DESeq provides statistical routines for determining differential expression in digital gene expression data using a model based on the negative binomial distribution. The resulting *p*-values were adjusted using the Benjamini and Hochberg’s approach for controlling the false discovery rate. Genes with an adjusted padj (p-adjusted) <0.05 found by DESeq were assigned as differentially expressed.

### GO and KEGG enrichment analysis of DEGs

Gene Ontology (GO) enrichment analysis of DEGs was implemented by the GOseq R package, in which gene length bias was corrected. GO terms with corrected *P*-values less than 0.05 were considered significantly enriched by differentially expressed genes. KEGG is a database resource for understanding high-level functions and utilities of the biological system, such as the cell, the organism and the ecosystem, based on molecular-level information, particularly large-scale molecular datasets generated by genome sequencing and other high-through put experimental technologies (http://www.genome.jp/kegg/). We used KOBAS software to test the statistical enrichment of DEGs in KEGG pathways.

### Novel transcripts prediction and differential alternative splicing analysis

The Cufflinks v2.1.1 Reference Annotation Based Transcript (RABT) assembly method was used to construct and identify both known and novel transcripts from TopHat alignment results. We used replicate multivariate analysis of transcript splicing (rMATS), which is a statistical method for detection of differential alternative splicing between two RNA-Seq samples.

### Validation of RNA-Seq by quantitative real-time PCR

Quantitative real-time PCR (qRT-PCR) was conducted using a Roche Light Cycler 480 system (Roche, Basel, Switzerland) in a final volume of 20 µl containing 10 µl THUNDERBIRD SYBR^®^ qPCR Mix (Toyobo, Japan), 1.6 µl of cDNA, 1.2 µl (6 pM) of the forward and reverse primers, and 6 µl of ddH_2_O. The qRT-PCR was performed using at least two independent biological replicates and three technical replicates of each biological replicate for each cDNA sample. The rice *Actin1* gene was used as the internal control gene. Relative gene expression levels were determined using the 2 (^−ΔΔCt^) method^83^. All the primer sequences used for qRT-PCR are provided in Supplementary Table S7.

### Data availability

Raw sequencing data have been deposited in the NCBI Gene Expression Omnibus (http://www.ncbi.nlm.nih.gov/geo) under the accession number GSE104928.

## Electronic supplementary material

Supplementary Table S1, S2, S7 and Supplementary Figure S1, S2

Supplementary Table S3

Supplementary Table S4

Supplementary Table S5

Supplementary Table S6
