# Supplementary material for: Transcriptome analysis of two contrasting rice cultivars during alkaline stress
Source: Sci Rep. 2018 Jun 25;8:9586. doi: 10.1038/s41598-018-27940-x (PMC6018505; doi:10.1038/s41598-018-27940-x)
Supplement: Supplementary file 1 — Supplementary Table S1, S2, S7 and Supplementary Figure S1, S2 [file 41598_2018_27940_MOESM1_ESM.pdf]

Transcriptome analysis of two contrasting rice cultivars during alkaline stress

Ning Li*, Hualong Liu*, Jian Sun*, Hongliang Zheng, Jingguo Wang, Luomiao Yang, Hongwei Zhao & Detang Zou

Rice Research Institute, College of Agriculture, Northeast Agricultural University, Harbin, China. Correspondence and requests for materials should be addressed to D.Z. (email: zoudtneau@126.com)

* These authors have contributed equally to this work.

Supplementary Table S1: Quality evaluation of RNA-seq data.

Sample name	Raw reads	Clean reads	clean bases	Error rate (%)	Q20 (%)	Q30 (%)	GC content (%)
CD_1	35106100	33222212	4.98G	0.01	97.79	94.45	54.88
CD_2	39051184	37404194	5.61G	0.01	97.72	94.36	53.33
CD_3	33963350	31829088	4.77G	0.01	97.38	93.57	55.46
CDT_1	37439328	35443102	5.32G	0.01	97.94	94.65	56.03
CDT_2	41797610	39746148	5.96G	0.01	97.91	94.62	57.64
CDT_3	41255270	40215320	6.03G	0.02	97.10	92.54	55.14
WD_1	27712092	26641752	4.00G	0.01	97.37	93.38	52.22
WD_2	27073366	26419246	3.96G	0.02	96.56	91.43	52.39
WD_3	36683258	33951874	5.09G	0.01	97.97	94.9	53.29
WDT_1	41979130	40621516	6.09G	0.02	97.03	92.43	56.30
WDT_2	48555630	46614884	6.99G	0.02	97.18	92.93	55.08

WDT_3	41811332	40259070	6.04G	0.02	97.05	92.64	55.52

Note: CD-1, CD-2, CD-3 and CDT-1, CDT-2, CDT-3 represent three biological replicates of control samples and alkaline stress samples of Caidao, respectively; WD-1, WD-2, WD-3 and WDT-1, WDT-2, WDT-3 represent three biological replicates of control samples and alkaline stress samples of WD20342, respectively.

Supplementary Table S2: Summary of Illumina transcriptome reads mapped to the reference genome.

Sample name	CD_1	CD_2	CD_3	CDT_1	CDT_2	CDT_3	WD_1	WD_2	WD_3	WDT_1	WDT_2	WDT_3
Total reads	33222212	37404194	31829088	35443102	39746148	40215320	26641752	26419246	33951874	40621516	46614884	40259070
Total mapped	28153042 (84.74%)	31205848 (83.43%)	25771632 (80.97%)	31302069 (88.32%)	33729487 (84.86%)	34938158 (86.88%)	22634771 (84.96%)	20826635 (78.83%)	27718761 (81.64%)	33657600 (82.86%)	39948998 (85.7%)	34475386 (85.63%)
Multiple mapped	408407 (1.23%)	429532 (1.15%)	473383 (1.49%)	410929 (1.16%)	354004 (0.89%)	408979 (1.02%)	345784 (1.3%)	308139 (1.17%)	427195 (1.26%)	463557 (1.14%)	515563 (1.11%)	401138 (1%)
Uniquely mapped	27744635 (83.51%)	30776316 (82.28%)	25298249 (79.48%)	30891140 (87.16%)	33375483 (83.97%)	34529179 (85.86%)	22288987 (83.66%)	20518496 (77.66%)	27291566 (80.38%)	33194043 (81.72%)	39433435 (84.59%)	34074248 (84.64%)
Reads map to '+'	13875644 (41.77%)	15384835 (41.13%)	12657030 (39.77%)	15440807 (43.57%)	16687874 (41.99%)	17268681 (42.94%)	11140408 (41.82%)	10251127 (38.8%)	13642233 (40.18%)	16626928 (40.93%)	19714016 (42.29%)	17028827 (42.3%)
Reads map to '-'	13868991 (41.75%)	15391481 (41.15%)	12641219 (39.72%)	15450333 (43.59%)	16687609 (41.99%)	17260498 (42.92%)	11148579 (41.85%)	10267369 (38.86%)	13649333 (40.2%)	16567115 (40.78%)	19719419 (42.3%)	17045421 (42.34%)

Non-splice reads	19536332 (58.81%)	22381203 (59.84%)	18845291 (59.21%)	20091997 (56.69%)	23211961 (58.4%)	23005958 (57.21%)	15803790 (59.32%)	15500230 (58.67%)	19215669 (56.6%)	23218409 (57.16%)	26818297 (57.53%)	23551291 (58.5%)
Splice reads	8208303 (24.71%)	8395113 (22.44%)	6452958 (20.27%)	10799143 (30.47%)	10163522 (25.57%)	11523221 (28.65%)	6485197 (24.34%)	5018266 (18.99%)	8075897 (23.79%)	9975634 (24.56%)	12615138 (27.06%)	10522957 (26.14%)

Note: CD-1, CD-2, CD-3 and CDT-1, CDT-2, CDT-3 represent three biological replicates of control samples and alkaline stress samples of Caidao, respectively; WD-1, WD-2, WD-3 and WDT-1, WDT-2, WDT-3 represent three biological replicates of control samples and alkaline stress samples of WD20342, respectively.

Supplementary Table S7: Primer sequences of qRT-PCR.

Primer Name		Primer sequence 5’-3’	Annealing temperature(℃)	Product size (bp)
LOC_Os02g49160	F	ACAAAGGTCTCGTCGCA	59	122
	R	CCCTCCTGGTCTTCGTAT		
LOC_Os02g24700	F	GTGCCGGTACGTGGTG	59	119
	R	GCAGGGAAGCGTGATG		
LOC_Os06g04590	F	GAGGAGTTCGGCTTCG	58	129
	R	CGGGTGCAGGAGTAGTG		
LOC_Os12g40900	F	GGAGACGCAGCAGAAGG	58	116
	R	CGGAGAAGCAGAGGAACA		
LOC_Os03g64260	F	AGGACCTGGGACCTGACT	59	116
	R	GGTGGCTTGGGCTTGTGC		
LOC_Os08g07100	F	GAAGACGATGAGCGAAAG	59	134
	R	GTAGGTGGCTGGTAACTGA		
LOC_Os08g04500	F	CTACTACCTGTGCCTACCTGC	58	138
	R	TTCCTCGGAAACATGAGC		
LOC_Os05g46460	F	CTCGTCATCGGATTGGC	59	108
	R	GGATTTGTGCGGGAGC		
LOC_Os08g30020	F	TCGGTGTTCTTTTCAATGTC	59	158
	R	GCTTTGCTGCCTCCCTTT		
LOC_Os12g26290	F	CTCTTCAAGCCTCGTGTT	60	128
	R	GGTGTTCAGGTAGAGCAAA		
Actin1	F	TGGCATCTCTCAGCACATTCC	55	150
	R	TGCACAATGGATGGGTCAGA		

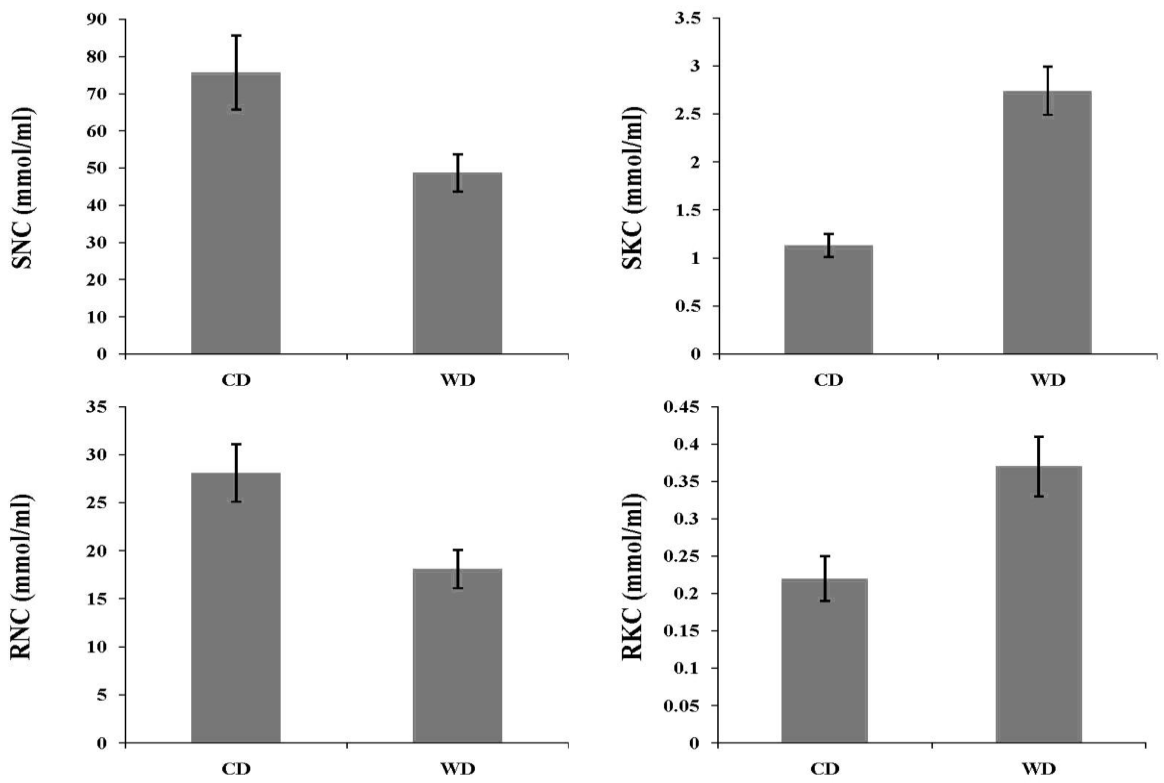

Supplementary Figure S1. Performance of alkaline-tolerant related traits for Caidao and WD20342. SNC and SKC mean that the concentration of Na^+ and K^+ in shoots, RNC and RKC mean that the concentration of Na^+ and K^+ in roots.

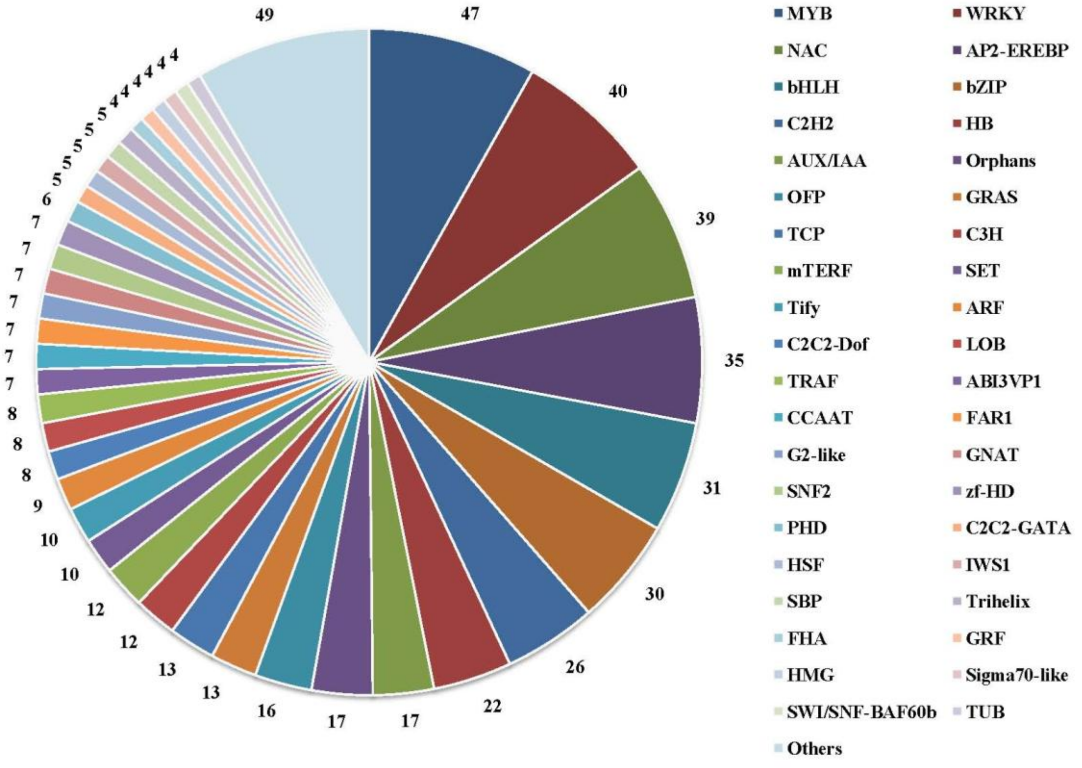

Supplementary Figure S2. Distribution of differentially expressed transcription factors in gene families.
